# GPs’ attitudes towards digital technologies for depression: an online survey in primary care

**DOI:** 10.3399/bjgp18X700721

**Published:** 2018-12-18

**Authors:** Josefien JF Breedvelt, Victoria Zamperoni, David Kessler, Heleen Riper, Annet M Kleiboer, Iris Elliott, Kathryn M Abel, Simon Gilbody, Claudi LH Bockting

**Affiliations:** Mental Health Foundation, London, and PhD candidate at Amsterdam UMC, Department of Psychiatry, University of Amsterdam, Amsterdam, the Netherlands.; Mental Health Foundation, London, UK.; Public Health Sciences, Bristol Medical School, Bristol, UK.; Clinical Psychology and eMental-Health, Faculty of Behavioural and Movement Sciences, Section of Clinical Psychology, Vrije Universiteit Amsterdam; Amsterdam UMC Department of Psychiatry; GGZ InGeest Specialised Mental Health Care, Amsterdam Public Health Research Institute, Amsterdam, the Netherlands.; Department of Clinical-Neuro-and Developmental Psychology, Vrije Universiteit Amsterdam, Amsterdam, the Netherlands.; Irish Human Rights and Equality Commission, Dublin, Ireland.; University of Manchester, and honorary consultant psychiatrist, Greater Manchester Mental Health NHS Foundation Trust, Manchester, UK.; University of York and Hull York Medical School, York, UK.; Psychiatry, and licensed clinical psychologist, Amsterdam UMC, Department of Psychiatry, University of Amsterdam, Amsterdam, the Netherlands.

**Keywords:** depression, general practice, mental health, primary health care, technological innovations

## Abstract

**Background:**

Digital or electronic mental health (e-mental health) interventions can be useful approaches in reducing the burden of depression, with tools available for use in prevention, treatment, or relapse prevention. They may have specific benefit for primary care, as depression is often managed in this setting. However, little is known about attitudes and barriers among GPs towards e-mental health interventions for depression.

**Aim:**

This study aimed to assess attitudes, knowledge, use, and barriers for depression-focused e-mental health among GPs across the UK.

**Design and setting:**

An online survey of self-selecting GPs in the UK conducted over a 10-day period in December 2017.

**Method:**

The survey consisted of 13 multiple choice questions posted on the Doctors.net.uk (DNUK) website.

**Results:**

In all, 1044 responses were included; 72% of GPs reported using at least one type of e-mental health intervention for depression. Overall, GPs reported that e-mental health interventions are most effective when delivered in a guided way, rather than in an unguided manner. In addition, 92% of GPs reported that neither they nor their colleagues received e-mental health training.

**Conclusion:**

A moderate number of GPs use e-mental health for depression in their services, and report it is likely that its use will increase. There is a gap in training and awareness of effective interventions. GPs consider guided e-mental health interventions to be most effective, in contrast to the unguided way it is mostly offered in primary care.

## INTRODUCTION

Depression is a leading cause of disability worldwide, the largest contributor to non-fatal health loss globally,^1^ and is associated with an increased risk of suicide.^2^ In England, it is estimated that 1.5 million people will have depression by 2026, resulting in societal costs of £12.2 billion a year to health care and social services, and in lost employment.^3^

In the UK, individuals with depression may receive treatment in primary care or be referred on to secondary or specialist services. For depression, GPs in England can refer on to an Improving Access to Psychological Therapies (IAPT)^4^^,^^5^ service. IAPT services, launched in 2008, aim to increase access to evidence-based treatments for anxiety and depression.^4^^,^^5^ Demand for IAPT services has increased over recent years, with referrals rising by 43% between 2012–2013 and 2014–2015, to a total of 1.2 million.^6^

Rising demand for services suggests that primary care will continue to be an increasingly major provider of mental health treatment for depression. Mental health problems have been cited as the second most common reason for primary care consultations in the UK, and GPs have reported spending an average of 30% of their time on mental health problems.^7^

Given increased pressure on services in the UK, it is important to explore new methods of managing depression that are effective for patients, and cost-effective for services. One such avenue is the use of electronic mental health (e-mental health) interventions. In England, the NHS has increasingly highlighted e-mental health in policy publications such as the *Five Year Forward View for Mental Health*,^4^ which includes technology-focused strategies to improve care.

Over the past decade, e-mental health interventions for depression have been used to expand support to a broader population.^8^ These interventions can be guided (supported by a therapist), or unguided (completed by the patient independently without therapist support). Computerised cognitive behavioural therapy (cCBT), in particular, can be a cost-effective approach, with meta-analyses indicating moderate effects on depression symptoms (severity measure for depression [SMD] = −0.35, 95% confidence interval [CI] = −0.57 to −0.12).^9^^,^^10^ cCBT has been recommended in guidance from the National Institute for Health and Care Excellence (NICE) for treatment of mild to moderate depression symptoms.^11^

**How this fits in**

Previous research has shown that guided e-mental health interventions may be effective for prevention, treatment, and management of depression. Little is known about the extent to which these tools are used in primary care in the UK, or about how GPs feel about such tools. Understanding GP knowledge and attitudes towards the use of e-mental health interventions will improve delivery of evidence-based e-mental health interventions for depression in primary care.

Studies show that the way in which e-mental health interventions are delivered may play a role in their impact. Evidence from a large UK randomised controlled study (REEACT1) found no additional benefit of offering unguided cCBT (with minimal telephone support) over usual GP care over the course of 12 months.^12^ Guided e-health interventions, however, appear to be more effective, and are associated with higher treatment adherence and efficacy.^13^^,^^14^

Despite ongoing research into effectiveness, little is known about the uptake of such interventions and the attitudes of UK professionals towards their implementation. Studies in other countries, or with other stakeholder groups, have found neutral to positive attitudes towards e-mental health interventions,^15^^,^^16^ with professionals feeling it could improve efficiency and quality of care.^17^ However, these studies also identified barriers, such as a lack of knowledge and awareness of e-mental health,^15^^,^^16^ the need for more information about the evidence base,^16^^,^^18^ lack of training,^17^ and ethical and clinical concerns.^16^^,^^18^

To the authors’ knowledge, no study has assessed GP awareness, attitudes, preferences, and current use of e-mental health for depression in the UK. This study aims to explore these topics in order to improve understanding of e-mental health implementation for depression in primary care.

## METHOD

The study is part of the European project eMEN, which aims to increase knowledge on e-mental health implementation, funded by the Interreg North West Europe Programme.^19^ The Mental Health Foundation (www.mentalhealth.org.uk), a UK-based mental health charity, led on survey design and quality assurance. Scripting was conducted with MedeConnect Healthcare Insight, the market research division of Doctors.net.uk (DNUK; www.doctors.net.uk). The survey was composed of 13 multiple-choice questions developed for this specific study in consultation with an advisory group of GPs, academics, and digital mental health experts. The 13 questions formed part of a monthly omnibus survey hosted by DNUK.

The link to the survey was displayed on the homepage of the DNUK website in November 2017. It was visible to all practising GPs who were members of DNUK and were active on the site (logged into their account) during the survey window. Four email reminders were circulated during the survey window. At the time of the survey, 45 154 GPs were members of DNUK, representing approximately 88% of UK GPs.^20^^–^23 Membership of DNUK requires a General Medical Council (GMC) registration number, ensuring responders are registered practitioners.^24^ An incentive of 2000 eSR points was offered for taking part in the survey (2000 points is equivalent to £10 and can be exchanged for high street vouchers or a charitable contribution). Responders were permitted to complete the survey once. Data collection and processing were compliant with the Market Research Society (MRS) code of conduct, and the legal and ethical framework of the British Healthcare Business Intelligence Association (BHBIA).^25^^,^^26^ To ensure regional representation, quotas were applied based on most recent GP workforce data. After a quota was filled, the survey was closed to that area to avoid further access from GPs in that location and avoid overrepresentation. GPs who started the survey while the quota was reached were able to continue. Any completed responses in excess of the quota were included in the analysis sample.

Survey data were analysed using descriptive statistics, with non-parametric tests employed to assess differences where descriptive results indicated this might be the case. IBM SPSS statistics software, (version 24) was used.

## RESULTS

Of 9928 individuals active on DNUK during the time of the surveys, 1260 persons accessed it. Responses were deemed eligible for analysis if the responder was:

- a principal GP (part owner of practice) salaried GP (employee of practice), GP registrar (junior doctor training in GP surgery), or locum GP (paid by session);
- based in the UK;
- had fully completed the survey; and
- the location quota had not reached capacity.

This resulted in 1044 eligible responses.

### Responder characteristics

Table 1 displays responder characteristics. Most were principal GPs (61%), male (56%), and aged 30–49 years old (68%). Responders qualified between 1968 and 2013. Based on 2017 data from the GMC, males were slightly over-represented (56%) compared to UK figures (47%).^27^

**Table 1.** Demographic characteristics of GP responders (*N* = 1044)

	***n***	**%**
**Age, years**		
30–39	299	29
40–49	410	39
50–59	260	25
≥60	75	7

**Sex**		
Male	584	56
Female	460	44

**GP type**		
Principal	634	61
Salaried	254	24
Locum	156	15

Based on 2017 data from NHS Digital, there was a slight over representation of GPs aged 40–49 years (39%) compared to English figures (27%); however, there was a similar proportion of GPs aged 30–39 years (29% in this survey compared to 30% nationally), 50–59 years (25% compared to 24%), and ≥60 years (7% compared to 8%).^20^

Table 2 shows GP practice characteristics. The regional distribution of responders was broadly representative of UK-wide GP membership. According to available national estimates, 81% (*n* = 41 817) of GPs were based in England^20^ compared to 80% (*n* = 833) in the present sample, 10% (*n* = 4920) in Scotland^21^ compared to 11% (*n* = 110) in the present sample, 6% in Wales (*n* = 2936)^22^ compared to 6% (*n* = 66) in the present sample, and 3% (*n* = 1722) in Northern Ireland^23^ compared to 3% (*n* = 35) in the present sample.

**Table 2.** GP practice characteristics (*N* = 1044)

	***n***	**%**
**Region**		
England	833	80
Scotland	110	11
Wales	66	6
Northern Ireland	35	3

**Setting**		
Urban	428	41
Suburban	264	26
Semi-rural	258	25
Rural	90	9
Other	4	0.4

**Practice size**	**Mean (SD)**	**Range**
Patients, *n*	9773 (5845)	500–62 000
GPs, *n*	7 (4)	1–40

SD = standard deviation.

### Current and future use of e-mental health interventions

In all, 72% of GPs (*n* = 756) endorsed using at least one type of e-mental health intervention for depression; 28% (*n* = 288) reported that they were not currently using e-mental health interventions for depression.

Variations in response by sex and age were reviewed (Table 3). χ^2^ tests revealed significant differences in reported use of e-mental health by age and by sex.

Younger GPs and female GPs were more likely to report using at least one type of e-mental health intervention (χ^2^ = 18.4, *P*<0.001, and χ^2^ = 13.0, *P* = 0.001, respectively). However, in both cases this difference had a small effect (*V* = 0.13 and φ = 0.11, respectively).

**Table 3.** GP use of e-mental health interventions, by age and sex

	**Not using any e-mental health intervention, *n* (%)^a^**	**Using at least one e-mental health intervention, *n* (%)^a^**
**Age, years**		
30–39	56 (19)	243 (81)
40–49	120 (29)	290 (71)
50–59	85 (33)	175 (67)
≥60	27(36)	48 (64)

**Sex**		
Male	187 (32)	397 (68)
Female	101 (22)	359 (78)

Total	288 (28)	756 (72)

a Percentages are for row totals and thus represent the % within each age band and sex that reported the use of at least one e-mental health tool versus no use.

A total of 73% (*n* = 758) of responders reported their practice’s use of e-mental health interventions will increase ‘somewhat’ or ‘substantially’ in the near future.

### Awareness of e-mental health interventions

The most widely known e-mental health intervention for depression was cCBT, with 47% (*n* = 491) of GPs reporting using cCBT in their practice, and 34% (*n* = 356) reporting they were aware of, but not currently using it (Table 4).

**Table 4.** GP use and awareness of e-mental health interventions for depression

**Type of e-mental health intervention**	**Not aware, *n* (%)**	**Aware but not using, *n* (%)**	**Using, *n* (%)**
Computerised cognitive behavioural therapy (cCBT)	197 (19)	356 (34)	491 (47)
Questionnaire for screening, assessment, or diagnosis	426 (41)	347 (33)	271 (26)
Mindfulness-based cognitive therapy	482 (46)	312 (30)	250 (24)
Psycho-education	608 (58)	223 (21)	213 (21)
Self-management	693 (66)	207 (20)	144 (14)
Active monitoring	693 (66)	269 (26)	82 (8)
Peer support	714 (68)	250 (24)	80 (8)
Sleep management	783 (75)	192 (18)	69 (7)
Behavioural activation	832 (80)	174 (17)	38 (3)

Digital questionnaires and digital mindfulness-based cognitive behavioural therapy (MBCT) were the second and third most commonly known and used interventions. Despite this, 41% (*n* = 426) and 46% (*n* = 482) of GPs, respectively, had never heard of these interventions. The least known intervention was digital behavioural activation, with only 21% (*n* = 212) reporting they were aware of, or used, the intervention.

There was an option to specify additional interventions in a free-text field. Most participants who used this replied ‘N/A’, with the remainder naming programmes that fall under existing categories (for example, ‘beating the blues’, a cCBT intervention, or ‘Big White Wall’, a peer support forum).

### Training in e-mental health interventions for depression

A total of 91.5% (*n* = 955) of GPs reported that neither they nor their colleagues had undertaken training in digital mental health, or that they were unsure if anyone had received training. Of the minority who reported training in their practice, most reported that they or their GP colleagues received the training (*n* = 80), with only 2% (*n* = 22) reporting that a practice nurse or other non-clinical colleague received training.

A χ^2^-squared test indicated there were significant regional differences in the proportion of GPs reporting any staff had received training versus none, or unknown levels of training (χ^2^ = 13.9, *P* = 0.003), with Northern Ireland reporting a higher proportion of trained staff (Table 5). However, this difference only had a small effect (*V* = 0.12).

**Table 5.** Staff with e-mental health training

	**England, *n* (%)^a^**	**Scotland, *n* (%)^a^**	**Wales, *n* (%)^a^**	**Northern Ireland, *n* (%)^a^**	**Total, *n* (%)**
Any staff trained	60 (7)	14 (13)	7 (11)	8 (23)	89 (9)
No staff trained/unsure	773 (93)	96 (87)	59 (89)	27 (77)	955 (92)

a The percentages are for column totals and thus represent the % within each nation that are trained versus untrained. Due to rounding, percentages may sum to more than 100.

### Implementation of e-mental health interventions

Across all therapeutic options, GPs indicated that six out of 10 e-mental health interventions for depression are currently predominately offered unguided, with only one intervention (digital questionnaire for screening/assessment) predominately offered guided, and the remaining three interventions offered as a mix of guided and unguided, dependent on patient need. When considering how interventions should be offered, ‘guided’ was the most frequent response for six of the 10 interventions, with ‘no strong opinion’ as the most frequent response for the remaining four interventions. Comparisons between preference and current use for the most commonly used interventions are outlined in Table 6.

**Table 6.** Current versus preferred implementation for commonly used e-mental health interventions

**Intervention**	**Guided**		**Unguided**		**Both/no preference**	
	**Current, *n* (%)**	**Preferred, *n* (%)**	**Current, *n* (%)**	**Preferred, *n* (%)**	**Current, *n* (%)**	**Preferred, *n* (%)**
cCBT, (*n* = 491)	92 (19)	260 (53)	215 (44)	52 (11)	184 (37)	179 (36)
Digital questionnaire, (*n* = 271)	88 (32)	117 (43)	83 (31)	46 (17)	100 (37)	108 (40)
Mindfulness-based cognitive therapy (*n* = 250)	31 (12)	118 (47)	122 (49)	28 (11)	97 (39)	104 (42
Psycho-education, (*n* = 213)	26 (12)	83 (39)	110 (52)	39 (18)	77 (36)	91 (43)
Self-management, (*n* = 144)	13 (9)	52 (36)	76 (53)	26 (18)	55 (38)	66 (46)

Responses were limited to participants who endorsed using each intervention, therefore the total number of responses will vary between each intervention type. cCBT = Computerised cognitive behavioural therapy.

### Barriers and benefits

Most responders rated all barriers in the survey as ‘somewhat significant’ (mode = 4). Barriers included infrastructure requirements; capacity; privacy concerns; awareness; availability of services; confidence to prescribe e-mental health interventions; willingness of patients; clinical risk; patient familiarity with and access to technology; uncertainty about the evidence base; medicolegal responsibility; and costs. There was an option to specify additional barriers in a free-text field. Most entries were ‘N/A’ or fell into existing categories. A few responses indicated e-mental health interventions were not deemed to be an appropriate substitute for face-to-face support.

‘Reduction in waiting times’ (53.8%, *n* = 563) and ‘service that supports more people experiencing symptoms of depression’ (62.4%, *n* = 651) were reported as the most likely benefits of implementing cCBT. One in 10 responders (10.7%, *n* = 112) reported that there would not be any benefit to implementing cCBT.

## DISCUSSION

### Summary

To the authors’ knowledge, the present study is the first to capture the views and attitudes of UK GPs towards the use of e-mental health interventions for depression in primary care.

This study found moderate use of e-mental health interventions among GPs. However, a majority (92%) of GPs were not aware of, or did not have, any e-mental health training for themselves or staff at their practice.

The study also revealed that, where e-mental health interventions for depression are offered, they are predominately offered unguided, despite GPs’ views that the most effective way to deliver such interventions would be with guidance.

GPs reported significant barriers to the implementation of e-mental health interventions, with all barriers in the survey rated as ‘somewhat significant’ by a majority of responders. Barriers included: infrastructure requirements; capacity; privacy concerns; awareness; availability of services; confidence to prescribe e-mental health interventions; willingness of patients; clinical risk; patient familiarity with and access to technology; uncertainty about the evidence base; medicolegal responsibility; and costs of implementation.

### Strengths and limitations

A strength of the study was its large sample size, which was broadly representative of the regional and age distribution of GPs. In addition, to the authors’ knowledge, this is the first survey of its kind conducted on e-mental health in the UK. But the study is limited by its self-selecting design in two ways. First, only GPs on DNUK were able to access the survey. Second, only 1260 of 9928 active users accessed it. This implies that the survey may not be representative of the views of all UK GPs, which affects the generalisability of these findings. However, individuals active on DNUK and who chose to complete the survey may have been more engaged with technology, and therefore more open to, or aware of, current e-mental health interventions.

Furthermore, due to the online survey structure, items had existing pre-defined responses. Although participants were offered free-text response options, these were not widely completed and may have limited the responses more than a qualitative approach would have done.

Additionally, due to the range of terms currently used in the field (for example, e-mental health, e-technologies, mHealth, digital, and so on), participants may have misunderstood some items, potentially biasing responses. It is recommended that future studies should not assume awareness of the range of interventions, and that definitions are provided where possible.

### Comparison with existing literature

All barriers listed in the survey were seen by GPs as ‘somewhat significant’, including infrastructure requirements, capacity, confidence to prescribe e-mental health interventions, willingness of patients, and uncertainty about the evidence base. This mirrors the findings from a previous survey conducted across a range of countries and stakeholders, which found that perceived barriers included concern about capacity for implementation, an expectation of negative attitudes from practitioners and patients, and concerns about effectiveness.^16^ However, although the same survey found cost-effectiveness to be the most significant perceived advantage of e-mental health technology, cost was cited as a ‘somewhat significant’ barrier by most GPs in the current study. This suggests that although there are shared challenges to implementing e-mental health interventions across stakeholders and countries, some barriers may be unique to specific settings.

### Implications for research and practice

The reported lack of awareness of current e-mental health interventions may be due in part to the limited amount of training and resources tailored to e-mental health interventions in primary care. Although e-learning modules for GPs are available,^28^ they focus on education about mental health rather than use of e-mental health interventions. As a result, it can prove challenging for GPs to decide which interventions to recommend and how to deliver them effectively. Thus, there may be a need for a clinical curriculum on e-mental health, alongside further training for existing staff, including GPs, nurses, assistants, and other healthcare professionals.

Such programmes should focus on specific audiences to ensure information is relevant to staff in a climate of increased pressure on services. A valuable first step might be to signpost to current initiatives like the NHS digital app library.^29^ Allowing other staff, such as trained nurses or lay counsellors, to support in the guidance of e-mental health interventions may also aid in alleviating barriers around capacity to implement interventions. However, further evaluation of effective models of guidance is required.

There is also a need for further evaluation of the relative effectiveness of different e-mental health interventions. This will aid in a greater understanding of what works in primary care and assist GPs and affiliated health staff in making informed decisions about appropriate tools to prescribe. This is supported by the rating of ‘concerns about the evidence base’ as a significant barrier by a large proportion of GPs in this survey.

It is recommended that future training for GPs captures this complexity, and that future research addresses concerns regarding implementation and effectiveness. This will ultimately improve support for prevention and treatment of depression in primary care.
